# ABCG2 gene polymorphism rs2231142 is associated with gout comorbidities but not allopurinol response in primary gout patients of a Chinese Han male population

**DOI:** 10.1186/s41065-019-0103-y

**Published:** 2019-07-24

**Authors:** Keke Zhang, Changgui Li

**Affiliations:** 1 Qingdao University, 38 Ningxia Road, Qingdao, 266003 China; 2 Institute of Clinical Research, The Affiliated Hospital of Qingdao University, 16 Jiangsu Road, Qingdao, 266003 China

**Keywords:** Gout, ABCG2, Polymorphisms, Allopurinol

## Abstract

**Background:**

One common ATP-binding cassette subfamily G member 2 (ABCG2) gene variant, which is encoded by the single nucleotide polymorphism (SNP) rs2231142, was identified to take an essential part in gouty arthritis. However, the relationship between rs2231142, gout comorbidities and therapeutic effect of allopurinol in Chinese Han male population is still unclear. Wherefore, this study explored into the association between ABCG2 SNP rs2231142 affecting common comorbidities and the therapeutic effect of allopurinol in Chinese Han male gout patients.

**Results:**

ABCG2 SNP rs2231142 and the gout comorbidities including nephrolithiasis and CKD were associated (*P* = 0.014 and *P* = 0.026). Group CKD stage = 1 were significantly different from those in group CKD stage≥2 regarding genotypes of ABCG2 gene polymorphism, while they were not significantly different from those in group CKD stage≥3. Meanwhile, the genotypes of rs2231142 and allopurinol response were not significantly associated (*P* = 0.588).

**Conclusions:**

ABCG2 rs2231142 may predict the risk of kidney comorbidities for Chinese Han male gout patients, but not allopurinol response.

## Background

As the characteristic illness resulting from symptomatic hyperuricemia, gout, the most common inflammatory arthritis for male especially in Western countries such as the United States, causes most of its patients to suffer acute attack including severe joint pain with sudden onset, fever, redness and swelling. Besides, the occurrence of Gout had remarkable increase worldwide in the last several decades and was recently reported as around 3–5% in some Asia-Pacific countries such as New Zealand and some regions of China [1–3]. The signature acute attacks of gout usually occur intermittently, especially in the early stages. And some patients are particularly vulnerable to recurrent gout due to predisposing systematic conditions. It is indicated that gout is not only limited to inflammation and other local symptoms in certain body parts such as the tissue around the joint,, but also associated with systematic disorders, for example, several cardiovascular-metabolic conditions [4, 5]. In the US National Health and Nutrition Examination Survey (NHANES) 2007–2008, it stated that the prevalence of coronary artery disease in gout patients is 25%, while that of hypertension is 74%, and the prevalence of diabetes is 26%, which are all higher than the corresponding data in patients without gout (5, 29, 7.8%, respectively) [6]. Furthermore, the therapeutic method to reduce serum uric acid (SUA) for gout patients and its reno-protective effect is under study [7].

To distinguish between primary gout and secondary gout, the primary gout is commonly defined as the direct result of underexcretion or overproduction of uric acid; in contrast, secondary gout is resulted from predisposing medications or conditions that cause hyperuricaemia. It was recognized that, excessive intake of certain foods (which include sea food and alcohol) was the predominant risk factor of primary gout, which was also closely correlated with obesity. The other common risk factors for primary gout include various metabolic syndromes, which cannot directly cause hyperuricemia [8].

Meanwhile, genome-wide association studies (GWAS) and meta-analyses were used to identify the genetic risk factors for gout, which were expected to be significant clinical indicators [9]. The predisposing genetic factors may influence prognosis, drug response and the risk of concomitant adverse events for individual patient. One major class of candidate makers is the variants of the gene encoding ATP-binding cassette (ABC) efflux drug-transporter. Initially, the ABC family was studied as the efflux transporter responsible for multidrug resistance in tumor cells [10] and was verified to regulate drug disposition in various organs. One of the urate efflux transporter, named ATP-binding cassette subfamily G member 2 (ABCG2), was discovered to be expressed on small intestine apical membrane and colonic epithelium apical membrane, as well as on the apical membrane of the proximal tubule cells of the kidney and the tubular membrane of hepatocytes. However, its expression level in the kidney is relatively lower than that in the liver and small intestine [11, 12]. It was reported in previous literature that the function loss of ABCG2 caused by genetic mutation was closely related to gout and gouty arthritis [13]. A common variant of *ABCG2* gene, which is the single nucleotide polymorphism (SNP) rs2231142, was pointed out as the key factor to influence urate and allopurinol extra-renal clearance and result in metabolic abnormality [14, 15].

As a class of most commonly used oral urate-lowering agents, allopurinol is conventionally used to treat gout. According to the guidelines of the American College of Rheumatology (ACR) and The European League Against Rheumatism (EULAR), allopurinol is the drug of first choice for the treatment of gout, unless not tolerated or contraindicated [16]. However, some patients showed incompliance with allopurinol, i.e. failed to reach the SUA target even though adequate doses of allopurinol were administrated [17]. The typical limitation of the allopurinol therapy is the high variability in uricotelic response ranging from good clinical response up to showing no benefit, and remarkable risks of severe side effects such as allopurinol hypersensitivity syndrome (AHS) [18]. Up to about 42% of the patients using allopurinol monotherapy were unable to reach the recommended SUA target of ≤6 mg/dl as a treatment goal [19]. Recently, Roberts RL et al. [15] demonstrated that the minor allele of *ABCG2* rs2231142 significantly increased the risk of reduced response to allopurinol in New Zealand gout populations, while OR = 2.71 (1.70–4.48), *P* = 6.0 × 10^− 5^.

This study was designed for further investigation into the connection of the *ABCG2* SNP rs2231142 with common comorbidities of gout and with therapeutic effect of allopurinol in Chinese Han male patients who were diagnosed with gout primarily without predisposing diseases, especially cancer and renal dysfunction.

## Results

Totally 1205 gout patients were enrolled. Clinical features of all the patients at baseline were summarized in Table 1. For the examination of the effects of the *ABCG2* rs2231142 genetic polymorphism on gout and its comorbidities, levels of serum uric acid and other biochemical results were compared among different genotype groups at baseline. The mean values and standard deviations of these baseline parameters were shown in Table 1. Compared with the therapeutic goal of allopurinol administration, which is SUA of 6 mg/dL, this group had a much higher mean baseline SUA of 474 μmol/L (8.7 mg/dL), which was consistent with the previously reported values in uncontrolled gout [20]. There are significant differences in SUA and SCr amongst the three groups: patients with the TT genotype had higher levels than those of the groups with the TG and GG genotypes (*P* < 0.001 and *P* = 0.005, respectively; Table 1). However, the other parameters had no significant differences among the groups with GG, GT and TT genotypes.

**Table 1:** Associations of the single-nucleotide polymorphisms rs2231142 in *ABCG2* with the clinical features of the gout patients

Parameter	Baseline (mean ± SEM)	(1)GG	(2)TG	(3)TT	(1)vs.(2)vs.(3) F/x^2^, *P* value	Partialη^2^
Age	52.75 ± 12.88	52.74 ± 13.04	52.95 ± 12.77	52.35 ± 12.97	0.208,0.812	0.000
BMI (kg/m^2^)	27.16 ± 3.62	27.56 ± 3.58	27.07 ± 3.90	26.89 ± 3.03	2.765,0.063	0.005
SBP (mmHg)	137.53 ± 19.90	138.24 ± 19.21	137.13 ± 19.83	137.53 ± 20.79	0.289,0.749	0.001
DBP (mmHg)	89.48 ± 12.95	89.83 ± 12.62	89.10 ± 13.04	89.87 ± 13.13	0.450,0.638	0.001
FPG (mmol/L)	6.19 ± 1.70	6.18 ± 1.69	6.22 ± 1.77	6.11 ± 1.45	0.435,0.647	0.000
Triglyceride (mmol/L)	2.45 ± 2.20	2.49 ± 2.25	2.41 ± 2.22	2.38 ± 1.56	0.234,0.791	0.001
Total cholesterol (mmol/L)	5.25 ± 1.19	5.29 ± 1.14	5.21 ± 1.23	5.29 ± 1.14	0.641,0.527	0.001
SUA (μmol/L)	474.51 ± 111.37	466.37 ± 115.83*	464.58 ± 112.59*	496.99 ± 104.43	8.798,< 0.001	0.014
Urea nitrogen (mmol/L)	5.81 ± 2.83	5.80 ± 1.92	5.78 ± 3.07	5.93 ± 3.29	0.259,0.771	0.001
SCr (μmol/L)	90.75 ± 42.39	87.18 ± 24.90*	89.13 ± 31.42*	97.74 ± 67.69	5.400,0.005	0.010

Data are the mean ± SEM. Statistical significance was defined as *P* < 0.05. Partial η^2^ is effect size. β = 0.2

*BMI* body mass index, *SBP* systolic blood pressure. *DBP* diastolic blood pressure, *FPG* fasting plasma glucose, *SUA* serum uric acid, *SCr* serum creatinine

The association between the SNP rs2231142 and the common comorbidities of gout was analyzed and the results were listed in Table 2. The frequency of nephrolithiasis for the patients with the TT genotype is less than that with the GG and TG genotypes, and the difference showed obvious statistical significance, as *P* = 0.006 and OR = 0.54 (0.35–0.84) for GG vs. TT, *P* = 0.012 and OR = 0.60 (0.41–0.90) for TG vs. TT, and *P* = 0.014 for the comparison among the three groups. In addition to that, the significant difference was also observed in the frequencies of CKD (*P* = 0.026), which had increased frequency for TT patients compared with the other two genotypes as illustrated in Table 2. In addition, for GG vs TT, OR = 1.54 (1.11–2.14); for TG vs. TT, OR = 1.39 (1.03–1.86). Except for nephrolithiasis and CKD, none of the other listed comorbidities were found to be associated with different genotypes of rs2231142, suggesting a unique association between the SNP and renal comorbidities. Thus, additional analysis was included to further explore the associations between rs2231142 and CKD stages. Among these individuals with gout, 540 patients had chronic kidney disease stage =1, 104 patients had chronic kidney disease stage≥2, as well as 13 patients chronic kidney disease stage≥3. The results listed in Table 2 showed the differences in the occurrences of CKD stage = 1 and those in group CKD stage≥2 were statistically significant regarding genotypes of *ABCG2* gene polymorphism (*P* < 0.001 and *P* = 0.026); whilst the difference was not statistically significant in group CKD stage≥3.

**Table 2:** Associations of the single-nucleotide polymorphisms rs2231142 in *ABCG2* with comorbidities of the gout patients

Comorbidities (with/without)		(1) GG	(2) TG	(3) TT	(1)vs.(2)vs.(3) F/x^2^, *P* value	(1)vs.(2) F/x^2^, *P* value	(1)vs.(2) OR (95% CI)	(1)vs.(3) F/x^2^, *P* value	(1)vs.(3) OR (95%CI)	(2)vs.(3) F/x^2^, *P* value	(2)vs.(3) OR (95%CI)
Hypertention	W/O^a^	198/110	340/226	175/120	1.952,0.377	–	1.19	–	1.08	–	0.91
(*n* = 713)	%^b^	64.3	60.1	59.3			(0.91–1.44)		(0.79–1.48)		(0.69–1.20)
Diabetes	W/O	48/270	109/471	54/241	2.033,0.362	–	0.95	–	1.23	–	1.29
(*n* = 211)	%^b^	15.1	18.8	18.3			(0.60–1.51)		(0.70–2.15)		(0.79–2.12)
Hyperlipidaemia	W/O	215/106	387/202	206/95	0.684,0.711	–	1.17	–	1.39	–	1.2
(*n* = 808)	%^b^	67.0	65.7	68.4			(0.87–1.59)		(0.91–1.99)		(0.86–1.63)
Obesity	W/O	234/63	411/140	209/73	2.276,0.320	–	1.44	–	1.75	–	1.21
(*n* = 854)	%^b^	78.8	74.6	74.1			(1.09–1.90)		(1.26–2.43)		(0.90–1.64)
Tophi	W/O	62/214	116/414	68/202	1.137,0.566	–	1.02	–	0.84	–	0.83
(*n* = 246)	%^b^	22.5	21.9	25.2			(0.71–1.44)		(0.57–1.25)		(0.59–1.17)
Nephrolithiasis	W/O	62/231	109/450	39/267	8.495,0.014	0.331,0.565	0.78	7.561,0.006	0.54	6.361,0.012	0.60
(*n* = 210)	%^b^	21.2	19.5	12.7			(0.55–1.12)		(0.35–0.84)		(0.41–0.90)
CKD	W/O	159/145	306/251	179/106	7.291,0.026	0.550,0.459	0.90	6.637,0.010	1.54	4.781,0.029	1.39
(*n* = 854)	%^b^	52.3	54.9	62.8			(0.68–1.19)		(1.11–2.14)		(1.03–1.86)
CKD stage = 1 (*n* = 540)		134	259	147	52.478,< 0.001						
	%^c^	44.1	46.5	51.6							
CKD stage≥2 (*n* = 104)		25	47	32	7.288,0.026						
	%^c^	8.2	8.4	11.2							
CKD stage≥3 (*n* = 13)		2	4	7	2.923,0.232						
	%^c^	0.6	0.7	2.5							

*CI* confidence interval, *OR* odds ratio

^a^ The number of the patients of the same genotype with/without the comorbidity (Patients with uncertain status of the comorbidity were excluded)

^b^ The frequency of the comorbidity in the patients with the same genotype (Patients with uncertain status of the comorbidity were excluded)

^c^ The frequency of CKD at a certain stage in the patients with the same genotype (Patients with uncertain status of CKD were excluded)

ABCG2, in addition to its function as UA transporter, is alternatively known as Breast Cancer Resistance Protein (BCRP) [21]. In view of the relevance of BCRP, the relevance of rs2231142 to the allopurinol reaction was also assessed. There were 922 gout patients received allopurinol treatment, 369 (40.0%) patients of whose had good compliance with treatment, while 273 (29.6%) of whose had poor compliance with treatment. Another 280 people cannot be classified as the allopurinol dose did not reach 300 mg per day or above. However, *ABCG2* SNP rs2231142 and allopurinol response was not significantly associated, because the odds ratio (OR with 95% confidence intervals) was 1.256 (0.813–1.941) between the groups with phenotype GG and TT, and OR was 1.132 (0.774–1.657) between the groups with phenotype TG and TT, respectively, *P* = 0.588 (Table 3).

**Table 3:** Genotype distributions of good responders and bad responders carrying the ABCG2 rs2231142 variant

rs2231142	n	(1)GG	(2)TG	(3)TT	*x*^2^,P	(1) vs (3) OR(95%CI)	(2) vs (3) OR(95%CI)
Good responders	369	90 (24%)	178 (49%)	101 (27%)	1.062,0.588	1.256 (0.813–1.941)	1.132 (0.774–1.657)
Bad responders	273	75 (27%)	131 (48%)	67 (25%)			

## Discussion

Zhu et al. [6] recently found that gout comorbidities have increased prevalence. The prevalence of a specific comorbidity in gout population might be 2–3 times higher compared to that of the same disorder in the population without gout. ABCG2, a drug exporter BCRP, usually has its expressions on epithelial cells of renal tubules [22] and small intestine [23]. The previous genetic and functional analysis [24] revealed high capacity of ABCG2 as the urate exporter, and the reduction in its transport function due to SNP rs2231142, a relatively common variant. Recently, this dysfunctional SNP was revealed to result in hyperuricaemia and decreased intestinal urate excretion, according to research on *ABCG2*-knockout mice [25] and hyperuricaemic patients [26]. This variant was also verified to cause destabilized BCRP and decreased expressions on the plasma membrane [21], which then lead to BCRP-mediated oxypurinol and allopurinol efflux modulation. According to the original genome-wide replication studies and association scans, common variants in ABCG2 (i.e. rs10011796 and rs2231142) demonstrated the strongest associations with gout [27, 28]. To our knowledge, the research presented here is the first evaluation of the connection between the *ABCG2* rs2231142 SNP and gout comorbidities among the Chinese Han male population.

CKD, a severe disease with poor prognosis even leading to mortality, is generally considered as a remarkable gout comorbidity. Among all the US adults with gout, approximately 70% (5.5 million) had some degree of CKD [6]. This was in agreement with our findings in Chinese Han male patients, which indicated 56% of the participants showed an eGFR < 90 ml/min/1.73 m2. This study revealed that *ABCG2* SNP rs2231142 was associated with gout renal comorbidities including nephrolithiasis and CKD. Significant differences in nephrolithiasis were detected, i.e. patients with TT genotype showed less occurrence than those with GG and TG genotypes. Similarly, CKD also demonstrated trends towards such associations. The differences in genotype caused significant variation in the occurrence of CDK stage = 1 and CDK stage≥2.

Allopurinol, a purine analogue which is normally metabolized into oxypurinol, is now the first-line medication to prevent gout [20]. Previous research suggested that the human *SLC2A9* [29] or *URAT1* [30] variants might affect the responses to allopurinol treatment in gout patients. However, rs2231142 was still the candidate genetic factor associated to poor response to allopurinol of the most research interest. Although little has been reported on the molecular mechanism of *ABCG2* expression and allopurinol pharmacodynamics and pharmacokinetics, the association had been confirmed by previous studies.. A recent study focusing on the New Zealand population with gout confirmed that rs2231142 but not rs10011796 has a relationship with the efficacy of allopurinol monotherapy [15]. And Wallace et al. [31] had also confirmed association of ABCG2 rs2231142 with poor response to allopurinol based on this NZ study together with Lasso study [32]. Interestingly, the finding of our present study suggested that the *ABCG2* SNP and the response to allopurinol monotherapy were not associated in Chinese Han gout patients. Among 922 patients with gout who were administrated with allopurinol, 40.0% patients were good responders, and 29.6% were poor responders, but each genotype of rs2231142 showed no particular association with allopurinol response (*P* = 0.588). Compared with the previous studies which had opposite conclusion, although it was possible that the ethnicity of population was the cause for the inconformity, the limitations of this study might interfere the analysis as well. The most predominant limitation for this section might be the neglection of drug adherence during participants selection. Both the two previous studies mentioned above had confirmed drug adherence in the participants, therefore those conclusions were less vulnerable to interferences such as inconsistent doses of allopurinol and altered pharmacokinetics by impaired organ function. Even with confirmed drug adherence, it was suggested the metabolizing process and drug response of allopurinol would still be interfered by renal insufficiency [15]. Herein, the other limitation worth to consider is the stratification of patients according to renal function, although the patients with predisposing renal insufficiency or renal failure was excluded from this study. CKD at various stages might also mask the positive association between genetic factor and drug response. Therefore, in follow-up studies, if the assessment of association can be designed based on division of groups with different kidney functions, the association between rs2231142 and allopurinol response might be further revealed for clinical significance.

Previous meta-analysis across race groups (including 238 East Asians) showed that the effects of a specific variant of rs2231142 were consistent among all ethnic groups, whereas various SNPs in *ABCG2* would change the direction and strength of association with allopurinol responses [33]. Thus, although some authors identified *ABCG2* rs2231142 GG carriers for better compliance with allopurinol treatment in gout, results herein indicate no difference in allopurinol response for rs2231142 polymorphism genotypes. This result, although “negative”, will be useful for future meta-analysis studies.

Collectively, although the rs2231142 genotype and in group CKD stage = 1 and those in group CKD stage≥2 was statistically significant with a large sample, the work presented here used limited size of sample (especially in the group with an CKD stage≥3) which are from a single Chinese Han male population. Additionally, many individuals were unaware of the renal disease until advanced stages of gout. Therefore, it is necessary to replicate this investigation to other populations and on larger cohorts, to verify the influence of *ABCG2* SNP as a risky factor in prognosis of gout renal comorbidities.

## Conclusions

The results indicated that ABCG2 rs2231142 may predict the risk of specific kidney comorbidities for primary gout patients in a population of Chinese Han male, but not allopurinol response. In the larger gout populations, more research is therefore required, to integrate the effects of genetic and acquired risk factors on urate transport to more accurately predict and prevent acquired comorbidities (e.g., CKD, nephrolithiasis, hypertension). Additionally, further omics approaches (epigenetic, transcriptomics, and proteomics studies etc.) are suggested to fully reveal the molecular mechanism of the pathogenesis for gout, and to allow the development of more personalized and highly efficient treatment plans.

## Methods

### Individuals and study design

Totally 1205 newly diagnosed gout arthritis male patients without a history of any urate-lowering medications were enrolled from the outpatient clinics in the Affiliated Hospital of Qingdao University, China, for the project in 2014–2018, complying with the Declaration of Helsinki.. Those with secondary gout (induced by cancer, use of diuretics, or renal insufficiency) were excluded. All the patients were diagnosed in accordance with the criteria for gout of American College of Rheumatology [34], who were provided with information about gout and advice for the treatment, e.g. diet and exercise. This study was designed to combine clinical diagnoses, physical examinations, interviews, pharmacy utilization and various laboratory data.

### Anthropometric and biochemical measurements

During clinic interviews, all subjects were asked questions about the comorbidities of interest, e.g. hypertension, diabetes mellitus, hyperlipidaemia, obesity, tophi, nephrolithiasis and chronic kidney disease (CKD). The body mass index (BMI, kg/m2) was calculated for each patient. Additionally, systolic and diastolic blood pressure (BP, mmHg) were measured. Levels of fasting serum glucose (FPG, mmol/L), triglycerides (TG, mmol/L), total cholesterol (TC, mmol/L), SUA (μmol/L), and serum creatinine (SCr,μmol/L) were measured after a 12-h fast state (Model 200; Toshiba, Tokyo, Japan). Modification of Diet in Renal Disease (MDRD) equation was used to calculate the estimated glomerular filtration rate (eGFR) and wherefore to evaluate the renal function in gout patients [35]. The equation is: eGFR = 186 × (SCr/88.4)-1.154 × age − 0.203. The eGFR was classified as normal, mildly impaired (stage 1), moderately impaired (stage 2), and severely impaired (stage 3) according to the following cutoffs: ⩾90, 60 to 89, 30 to 59, and < 30 mL/min/1.73 m^2^, respectively [36]. A total of 642 patients were selected for this section, who had the mono-allopurinol prescription with an untreated SUA obtained at most 18 months before the prescription. Good response had the definition of SUA⩽6 mg/dl on allopurinol ⩽300 mg/d; while poor response had the definition of SUA⩾6 mg/dl despite allopurinol⩾300 mg/d [15]. Affymetrix Axiom Genome-Wide CHB Array was used to conduct genotyping.

### Statistical analysis

SPSS software (IBM Corporation, Armonk, USA) was used for all calculations and statistics. Data are presented in the format of mean ± SD or n (%). Differences among the three genotypes were analyzed with Kruskal-Wallis test or ANOVA followed by the Student-Newman-Keuls multiple range test. Incidence of comorbidities in gout patients was assessed with the Student t test for continuous data and chi-square test for categorical data. Linear regression was conducted to assess associations of variables with therapeutic effect of allopurinol and also associations between variables. Statistical significance was defined as *P* < 0.05 (two-tailed). The odds ratio (OR) values are presented with 95% confidence intervals (CIs).

## Data Availability

The original datasets used and analyzed in this study are available on reasonable request.

## Abbreviations

ABCG2: ATP-binding cassette subfamily G member 2
ACR: American College of Rheumatology
AHS: Allopurinol hypersensitivity syndrome
BCRP: Breast cancer resistance protein
BMI: Body mass index
BP: Blood pressure
CIs: Confidence intervals
CKD: Chronic kidney disease
eGFR: Estimated glomerular filtration rate
EULAR: European League Against Rheumatism
FPG: Fasting serum glucose
GWAS: Genome-wide association studies
HWE: Hardy–Weinberg equilibrium
NHANES: National Health and Nutrition Examination Survey
OR: Odds ratio
P: *P* value
SCr: Serum creatinine
SD: Standard deviation
SNP: Single nucleotide polymorphism
SUA: Serum uric acid
TC: Total cholesterol
TG: Triglycerides
URAT1: Urate transporter 1

## References

[1] Singh JA (2013). Racial and gender disparities among patients with gout. Curr Rheumatol Rep.

[2] Winnard D, Wright C, Jackson G (2012). Gout, diabetes and cardiovascular disease in the Aotearoa New Zealand adult population: co-prevalence and implications for clinical practice. NZ Med J.

[3] VanItallie TB (2010). Gout: epitome of painful arthritis. Metabolism.

[4] Singh JA, Strand V (2008). Gout is associated with more comorbidities, poorer health-related quality of life and higher healthcare utilisation in US veterans. Ann Rheum Dis.

[5] Kodama S, Saito K, Yachi Y, Asumi M, Sugawara A, Totsuka K, Saito A, Sone H (2009). Association between serum uric acid and development of type 2 diabetes. Diabetes Care.

[6] Zhu Y, Pandya BJ, Choi HK (2012). Comorbidities of gout and hyperuricemia in the US general population: NHANES 2007-2008. Am J Med.

[7] Kanji T, Gandhi M, Clase CM, Yang R (2015). Urate lowering therapy to improve renal outcomes in patients with chronic kidney disease: systematic review and meta-analysis. BMC Nephrol.

[8] MacFarlane LA, Kim SC (2014). Gout: a review of nonmodifiable and modifiable risk factors. Rheum Dis Clin N Am.

[9] Li C, Li Z, Liu S, Wang C, Han L, Cui L, Zhou J, Zou H. Genome-wide association analysis identifies three new risk loci for gout arthritis in Han Chinese. Nat Commun. 2015;6:7041. 10.1038/ncomms8041 PMC4479022 25967671

[10] Dietrich C, Geier A, Wasmuth H, Matern S, Gartung C, de Waart D, Elferink R (2004). Influence of biliary cirrhosis on the detoxification and elimination of a food derived carcinogen. Gut.

[11] Woodward OM, Köttgen A, Coresh J, Boerwinkle E, Guggino WB, Köttgen M (2009). Identification of a urate transporter, ABCG2, with a common functional polymorphism causing gout. Proc Natl Acad Sci.

[12] Mao Q, Unadkat JD (2015). Role of the breast cancer resistance protein (BCRP/ABCG2) in drug transport—an update. AAPS J.

[13] Matsuo H, Takada T, Ichida K, Nakamura T, Nakayama A, Ikebuchi Y, Ito K, Kusanagi Y, Chiba T, Tadokoro S (2009). Common defects of ABCG2, a high-capacity urate exporter, cause gout: a function-based genetic analysis in a Japanese population. Sci Transl Med.

[14] Matsuo H, Nakayama A, Sakiyama M, Chiba T, Shimizu S, Kawamura Y, Nakashima H, Nakamura T, Takada Y, Oikawa Y (2014). ABCG2 dysfunction causes hyperuricemia due to both renal urate underexcretion and renal urate overload. Sci Rep.

[15] Roberts R L, Wallace M C, Phipps-Green A J, Topless R, Drake J M, Tan P, Dalbeth N, Merriman T R, Stamp L K (2016). ABCG2 loss-of-function polymorphism predicts poor response to allopurinol in patients with gout. The Pharmacogenomics Journal.

[16] Neogi T, Jansen TLTA, Dalbeth N, Fransen J, Schumacher HR, Berendsen D, Brown M, Choi H, Edwards NL, Janssens HJ (2015). 2015 gout classification criteria: an American College of Rheumatology/European league against rheumatism collaborative initiative. Arthritis Rheumatol.

[17] Wright DF, Duffull SB, Merriman TR, Dalbeth N, Barclay ML, Stamp LK (2016). Predicting allopurinol response in patients with gout. Br J Clin Pharmacol.

[18] Stamp LK, Day RO, Yun J (2016). Allopurinol hypersensitivity: investigating the cause and minimizing the risk. Nat Rev Rheumatol.

[19] Becker MA, Schumacher HR, Espinoza LR, Wells AF, MacDonald P, Lloyd E, Lademacher C (2010). The urate-lowering efficacy and safety of febuxostat in the treatment of the hyperuricemia of gout: the CONFIRMS trial. Arthritis Res Ther.

[20] Khanna D, Fitzgerald JD, Khanna PP, Bae S, Singh MK, Neogi T, Pillinger MH, Merill J, Lee S, Prakash S (2012). 2012 American College of Rheumatology guidelines for management of gout. Part 1: systematic nonpharmacologic and pharmacologic therapeutic approaches to hyperuricemia. Arthritis Care Res.

[21] Woodward OM, Tukaye DN, Cui J, Greenwell P, Constantoulakis LM, Parker BS, Rao A, Köttgen M, Maloney PC, Guggino WB (2013). Gout-causing Q141K mutation in ABCG2 leads to instability of the nucleotide-binding domain and can be corrected with small molecules. Proc Natl Acad Sci.

[22] Huls M, Brown C, Windass A, Sayer R, Van Den Heuvel J, Heemskerk S, Russel F, Masereeuw R (2008). The breast cancer resistance protein transporter ABCG2 is expressed in the human kidney proximal tubule apical membrane. Kidney Int.

[23] Maliepaard M, Scheffer GL, Faneyte IF, van Gastelen MA, Pijnenborg AC, Schinkel AH, van de Vijver MJ, Scheper RJ, Schellens JH (2001). Subcellular localization and distribution of the breast cancer resistance protein transporter in normal human tissues. Cancer Res.

[24] Matsuo H, Ichida K, Takada T, Nakayama A, Nakashima H, Nakamura T, Kawamura Y, Takada Y, Yamamoto K, Inoue H (2013). Common dysfunctional variants in ABCG2 are a major cause of early-onset gout. Sci Rep.

[25] Ichida K, Matsuo H, Takada T, Nakayama A, Murakami K, Shimizu T, Yamanashi Y, Kasuga H, Nakashima H, Nakamura T (2012). Decreased extra-renal urate excretion is a common cause of hyperuricemia. Nat Commun.

[26] Matsuo H, Tsunoda T, Ooyama K, Sakiyama M, Sogo T, Takada T, Nakashima A, Nakayama A, Kawaguchi M, Higashino T. Hyperuricemia in acute gastroenteritis is caused by decreased urate excretion via ABCG2. Sci Rep. 2016;6:31003. 10.1038/srep31003 PMC5004129 27571712

[27] Köttgen A, Albrecht E, Teumer A, Vitart V, Krumsiek J, Hundertmark C, Pistis G, Ruggiero D, O'Seaghdha CM, Haller T (2013). Genome-wide association analyses identify 18 new loci associated with serum urate concentrations. Nat Genet.

[28] Okada Y, Sim X, Go MJ, Wu J-Y, Gu D, Takeuchi F, Takahashi A, Maeda S, Tsunoda T, Chen P (2012). Meta-analysis identifies multiple loci associated with kidney function-related traits in east Asian populations. Nat Genet.

[29] Anzai N, Ichida K, Jutabha P, Kimura T, Babu E, Jin CJ, Srivastava S, Kitamura K, Hisatome I, Endou H (2008). Plasma urate level is directly regulated by a voltage-driven urate efflux transporter URATv1 (SLC2A9) in humans. J Biol Chem.

[30] Iwanaga T, Kobayashi D, Hirayama M, Maeda T, Tamai I (2005). Involvement of uric acid transporter in increased renal clearance of the xanthine oxidase inhibitor oxypurinol induced by a uricosuric agent, benzbromarone. Drug Metab Dispos.

[31] Wallace MC, Roberts RL, Nanavati P, Miner JN, Dalbeth N, Topless R, Merriman TR, Stamp LK (2018). Association between ABCG2 rs2231142 and poor response to allopurinol: replication and meta-analysis. Rheumatology (Oxford).

[32] Becker M, Fitz-Patrick D, Choi H (2015). An open-label, 6-month study of allopurinol safety in gout: the LASSO study. Semin Arthritis Rheum.

[33] Wen C, Yee S, Liang X, Hoffmann T, Kvale M, Banda Y, Jorgenson E, Schaefer C, Risch N, Giacomini K (2015). Genome-wide association study identifies ABCG2 (BCRP) as an allopurinol transporter and a determinant of drug response. Clin. Pharmacol. Ther..

[34] Wallace SL, Robinson H, Masi AT, Decker JL, Mccarty DJ (1977). Preliminary criteria for the classification of the acute arthritis of primary gout. Arthritis Rheumatol.

[35] Levey AS, Bosch JP, Lewis JB, Greene T, Rogers N, Roth D (1999). A more accurate method to estimate glomerular filtration rate from serum creatinine: a new prediction equation. Ann Intern Med.

[36] Levey AS, Stevens LA, Schmid CH, Zhang YL, Castro AF, Feldman HI, Kusek JW, Eggers P, Van Lente F, Greene T (2009). A new equation to estimate glomerular filtration rate. Ann Intern Med.

